# Ectomycorrhizal communities of adult and young European larch are diverse and dynamics at high altitudinal sites

**DOI:** 10.1007/s11104-024-06721-8

**Published:** 2024-05-21

**Authors:** Edoardo Mandolini, Margit Bacher, Ursula Peintner

**Affiliations:** Department of Microbiology, University of Innsbruck, Technikerstrasse 25B, 6020 Innsbruck, Austria

**Keywords:** *Larix decidua*, Alpine larch, *Suillus*, Alps, Rare species turnover, Early-stage mycorrhizae, Host specialists

## Abstract

**Background/Aims:**

The European larch is a pioneer tree and a valuable economic resource in subalpine ecosystems, thus playing crucial roles to ecosystem services and human activities. However, their ectomycorrhizal fungal community remains unknown in high altitudinal natural habitats. Here, we explore the mycobiont diversity of *Larix decidua* var. *decidua* between naturally rejuvenated and adult trees, compare ectomycorrhizal colonization patterns in geographically disjunct areas within the Alps of South Tyrol, Italy, characterized by distinct climatic conditions, and explore turnover rates across various seasons.

**Methods:**

Our approach combines morphotyping of mycorrhized root tips with molecular analysis. Particular effort was given to monitor both ectomycorrhizal host-specialist and -generalist fungi.

**Results:**

Both adult and young trees show a 100% mycorrhization rate, with a total diversity of 68 ectomycorrhizal species. The ectomycorrhizal composition is dominated by typical host specialists of larch trees (e.g., *Lactarius porninsis*, *Russula laricina*, *Suillus cavipes*, *S. grevillei*, *S. viscidus*), which are widely distributed across sites. A rich diversity of host generalists was also detected. The composition of rare species within a habitat was comparatively consistent during one sampling campaign, but exhibited significant differences among individual sampling campaigns. The ectomycorrhizal compositions were only weakly correlated with distinct climatic conditions and tree ages. However, species richness and diversity, particularly of generalist fungi, was consistently higher in warmer, drier sites compared to cooler, more humid ones.

**Conclusions:**

This study suggests potential mycobiont community shifts across climatic conditions with significant implications for the adaptability and resilience of subalpine forests in the face of climate change.

**Supplementary Information:**

The online version contains supplementary material available at 10.1007/s11104-024-06721-8.

## Introduction

The European larch (*Larix decidua* Mill.) is an economically and traditionally important timber tree in Europe. Its fast-growing nature, high adaptability, and its hard, durable, and fragrant wood are widely recognized and appreciated qualities since the eighteenth century. The European larch occurs in the central and eastern mountains of Europe. Its ecological amplitude is comparatively large, as it can grow both in continental subalpine climates, tolerating very cold, dry, and snowy winters, and in sub-continental climates with mild temperatures. It has thus, a broad vertical range, forming forests between 180 m (in Poland) to 2500 m (central and south-western- Alps). This deciduous conifer is considered to be a pioneer tree, tolerating nutrient-poor ground, a range of pH from neutral to acid, and well-drained soils. Thus, it can initiate the process of reforestation by enriching the soil with organic matter or by promoting biodiversity (Da Ronch et al. 2016). These adaptations make larch particularly important at high-altitude ecosystem where harsh climatic conditions and soil erosion may limit tree establishment and forest development.

Three recognized varieties of *Larix decidua* exist, and stand in different geographical areas: the Alpine larch (*Larix decidua* var. *decidua*), the Carpathian larch (*Larix decidua* var. *carpatica*), and the Poland larch (*Larix decidua* var. *polonica*). In the Alps, Alpine larch forms the upper tree limit and can occur both in pure forests, such as in the Italian, French, and Southern Swiss Alps, or in mixed stands with other subalpine tree species, such as Swiss stone pine (*Pinus cembra*), green alder (*Alnus viridisi*), dwarf mountain pine (*Pinus mugo*), or Norway spruce (*Picea abies*). However, the projected rates of temperature rise and alterations in precipitation patterns are considered to pose significant threats to larch forests, especially in high altitudinal regions (Obojes et al. 2018; Sasani et al. 2021). Here, the ecosystem is particularly susceptible to the ongoing environmental changes and a feedback from the physiological responses of trees to the community composition of microorganisms in the soil, including fungi, is expected (Tedersoo et al. 2012, 2014; Vašutová et al. 2017). The fungal compositional changes in response to experimental warming have been investigated in different high altitudinal ecosystems (Schindlbacher et al. 2011, 2015; Treseder et al. 2016; Solly et al. 2017), but the intensity of such changes differed strongly among ecosystems. The impact of warmer and drier environmental conditions on the ectomycorrhizal fungal associated to tree roots remains largely unexplored in the natural environment, although basic and functional knowledge on these important mutualistic symbiont communities is urgently needed.

*Larix decidua* is, like all other coniferous trees of the Pinaceae, usually strongly mycorrhized (Bacher et al. 2010; Leski and Rudawska 2012). Ectomycorrhizal fungi (EcM) facilitate nutrient absorption and water uptake, increase resistance to fungal pathogens, and enhance stress tolerance of the plant host (Rincón et al. 2005). Compared to non-mycorrhized plants, mycorrhized individuals grow faster, have higher vigor, and form more biomass in shoots and roots (Göbl 1963; Piola et al. 1995; Ohga and Wood 2000). EcM fungi vary dramatically in enzymatic capabilities, environmental tolerance, and host specificity (Keller 1992; Agerer 2001; Talbot et al. 2013). Concerning host specificity, most EcM fungi live along a continuum between complete generalization to high degree of host specificity, where generalists form symbiosis with phylogenetically diverse array of plant host, while host specialists are exclusively associated with members of a specific tree family or genus (Pérez-Pazos et al. 2021). EcM specialists are usually physiologically optimally adapted to their host partner, resulting, for example, in a very efficient exchange of trace elements and carbohydrates (Bruns et al. 2002). Furthermore, host-specialist fungi possess more significant competitive advantages during host colonization, and thus have greater access to the resources of the host (Bruns et al. 2002). Host specialists typically dominate the EcM species found in high altitudinal forest habitats where environmental conditions limit tree growth (Moser 1958; Heumader 1992; Mandolini et al. 2022). The European larch is well-known for being associated with a range of host specialists: *Suillus bresadolae, S. cavipes. S. greivillei* and *S. tridentinus, Lactarius porninsis, Russula laricina, Tricholoma psammopus, Hygrophorus speciosus* and *H. lucorum* are the most important examples of *L. decidua* host-specialists (Smith 2017; Miyamoto et al. 2019). We must clarify that this rigorous specialization is solely limited to the fungal side, whereas the plant still possesses the capability to contemporarily associate with many fungal species. In fact, the potential drawbacks for a plant associating mainly with specialist fungi are limited adaptability to changing environmental conditions. These drawbacks can be compensated by generalist EcM present in the habitat, whose function ranges from trophic interactions to protective effects (Selosse et al. 2004), but they are usually occurring in low abundance only (Kranabetter 2004; Rainer et al. 2015).

Despite their ecological significance, the ectomycorrhizal or mycobiont diversity of *L. decidua* roots remains inadequately investigated and limited to specific circumstances. Previous studies primarily focused on either seedlings grown in bare-root forest nurseries (Leski et al. 2008; Bacher et al. 2010) or in young, naturally regenerated seedlings of Poland forests (Leski and Rudawska 2012). However, the mycobiont species composition usually changes during the tree development, and seedlings have a different species composition than adult trees (Visser 1995; Trocha et al. 2006; Bacher et al. 2010). It is also important to note that distinctions may exist in the composition of EcM species between nursery-cultivated seedlings and naturally established trees developing in their natural habitat (Southworth 2009), depending on nursery management practices (Repáč 2011, Menkis et al. 2005). In addition, many EcM fungi are observed rarely and sporadically because of the inherently limited sampling efforts challenged by the necessity of screening huge amounts of soil volumes (Taylor 2002). The dynamics of EcM communities in response to plant growth and fluctuating environmental conditions, whether across a single season or multiple years, remain poorly explored in *L. decidua* forests.

In high altitudinal natural environments, knowledge concerning EcM communities of *L. decidua* is restricted to either mycorrhizal morphotyping without use of molecular analysis in afforestation areas (Göbl and Ladurner 2000), or to data concerning fungal fruiting body occurrence (Moser 1956; Horak 1963, 1985). Thus, in this study, we aim to identify the EcM partners of *L. decidua* var. *decidua* across varying high altitudinal forests in the Alps of South Tirol, Italy. Specifically, we wanted to address the following questions: (i) Are there EcM fungal communities which are characteristic for *L. decidua* habitats in general, or are there fungal species adapted to specific habitat conditions? (ii) Are there distinct EcM fungal communities that are characteristic for the different tree age stages? (iii) Does the mycorrhizal composition of adult larches undergo any changes within a year or across two different years?

## Materials and methods

### Study sites

**Fig. 1:**
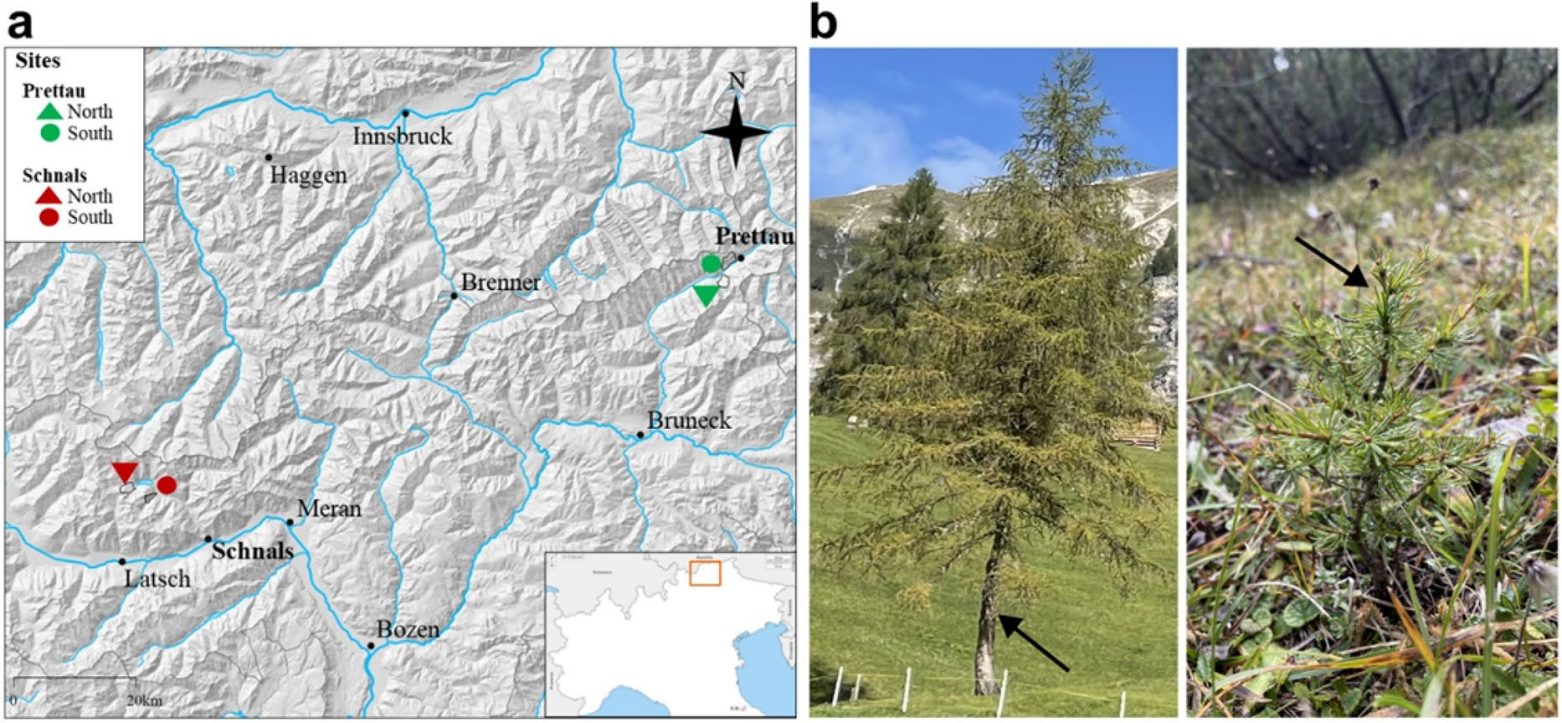
Study location of *Larix decidua* forests object of this study. (**a**) The two locations in South Tirol (Italy) with north- and south-exposed sites are marked. (**b**) Age stages of adult (left) and young (right) *L. decidua* individuals sampled

Two different locations in South Tyrol (Italy) were chosen to carry out the study on EcM fungal communities in *L. decidua* root systems, namely the area around the villages of Prettau and Schnals (Table 1, Fig. 1a). These locations are located in two different valleys within South Tyrol, Ahrntal and Schnalstal, respectively, that exhibit contrasting geographical features, resulting in distinct climatic conditions due to variations in wind exposure, annual rainfall, and historical development (Table 1). Particularly, Ahrntal has higher mean annual temperatures and lower annual precipitations than Schnalstal, resulting in an overall milder climate. In each valley, we selected two distinct high altitudinal sites of *L. decidua* forests: one situated on a south-exposed slope (S) and another on a north-facing slope (N). This determination was based on expert advice from staff of the Department of Agriculture and Forestry of South Tyrol Administration upon on-ground observation of local vegetation, soil moisture, and local climate. The orientation of the slopes plays a crucial role in determining the local microclimate, with southern slopes typically experiencing lower moisture levels and higher temperatures, while northern slopes tend to be cooler and more humid (data not shown). In Prettau, the *L. decidua* stands were interspersed with *Pinus cembra* and *Picea abies* individuals. In Schnals, only *P. cembra* trees were additionally present. A typical subalpine vegetation covered the forest ground at all sites. We also measured the soil pH at each site using a 0.01 M CaCl2 solution, as previously described (Thomas 1996). Snow typically covers the sampling areas from October or November until April or May in all locations.

**Table 1:** Study sites for the investigation of EcM communities associated to *Larix decidua* forests in South Tyrol, Italy

Location	Prettau		Schnals	
Exposure (sites)	N	S	N	S
Position	47.021360, 12.092171	47.040662, 12.097492	46.731987, 10.801964	46.716817, 10.850029
Altitude	1910 m	1760 m	1850 m	1860 m
pH	3.3	3.7	4.3	4.3
Soil type*	Semipodsole	Semipodsole	Semipodsole	Semipodsole
Temperature (°C)**	5		6	
Precipitation (mm)**	957		672	

*https://tirolatlas.uibk.ac.at/maps/interface/topo.py/index?image=c7_boden

**Annual mean; https://weather.provinz.bz.it/default.asp

### Sample collection

Sampling was carried out in an area within a range of ca. 2 km^2^ around the coordinates provided in Table 1. Importantly, all locations were sampled at the same elevation, roughly between 1700 and 1900 m above sea level. At all four sites, two different age stages of *L. decidua* were selected based on their height, stem diameter, and expert evaluation, namely adults (adult, 10–15 m high, > 50 years old) and naturally regenerated individuals (young, 6—15 cm tall with maximum stem diameter of 5–10 mm, < 3 years old) (Fig. 1b). When sampling the EcM fungal community residing in the root systems of adult larch trees, 5 soil blocks were randomly taken in the proximity of 5 adult individuals (ca. 1–2 m away from the trunk) from each site at intervals of about 6 months, to cover both spring and autumn, for two years (Table 2). The samples were taken with a spade, they had an area of 20 × 20 cm and varied in depth (10—20 cm deep), depending on the subsoil conditions. Particular care was taken to minimize proximity to nearby interspersed spruce or stone pine trees, when present. To determine the autochthonous EcM fungal communities of young larches, 5 whole individuals were extracted from each site each year in spring, for three years (Table 2). We retained to collect seedlings in autumn to avoid major natural disruption inherent to the removal of young individuals from a natural area.

**Table 2:** Sampling time for young and adult *L. decidua* trees in both locations. Monthly precipitations and temperatures (min–max) are also reported for each sampling time

Sampling dates					Precipitation (mm)*		Temperature (°C)**	
Tree age	Time	Month	Year	Season	Prettau	Schnals	Prettau	Schnals
Young	**II**	June	2007	Spring	123	79	6.9—18.8	7.4—17.4
	**IV**	June	2008	Spring	75	107	6.9—19.2	7.4—17.3
	**V**	June	2009	Spring	156	69	5.9—16	7—16.8
Adult	**I**	October	2006	Autumn	32	68	2.4—13.2	3.9—11.9
	**II**	June	2007	Spring	123	79	6.9—18.8	7.4—17.4
	**III**	October	2007	Autumn	106	5	-0.7—10.3	1.6—10.4
	**IV**	June	2008	Spring	75	107	6.9—19.2	7.4—17.3

*Average of the month; **Min–max of the month

### Mycorrhizal morphotyping, DNA extraction, and sequencing

After each sampling campaign, mycorrhizal morphotyping were performed as previously described (Mandolini et al. 2022). Briefly, root systems of the young larches and soil blocks were gently cleaned. From these, mycorrhized fine roots were randomly taken and counted for a total of 600 root tips from each age class, site, and time. All selected root tips were analyzed for morphological identification, that is distinguished based on morphological criteria (Agerer 2001) and classified into different morphotypes (MT).

DNA extraction (CTAB protocol) and molecular amplification (PCR reagent mix and conditions) from at least 4 root tips of each MT were performed as previously described (Bacher et al. 2010). The following primer combinations were used: ITS1F × LR15, ITS1F × LR21, ITS1F × NL4 and ITS1F × ITS4 for DNA amplification to account in intraspecific sequence polymorphism. Purified PCR products were sent to Genoscreen (Lille, France) for Sanger sequencing analyses starting from the beginning of the ITS1 region. Genomic DNA from MT which did not have at least 4 root tips was not extracted, thus not sequenced, and were categorized as “Rare MT”.

### Data analyses and statistics

Sequencing analysis, taxonomic assignment, and data analysis were performed as previously described (Mandolini et al. 2022). Briefly, resulting rDNA ITS sequences were edited and checked using Sequencer (v.4.6; Gene codes Inc. Ann Arbor, MI). Sequences were clustered into operational taxonomical units (OTUs) with a 97% score similarity. Taxonomic assignment was obtained using full UNITE + INSD Fungal ITS dataset 2 v.18–07–2023 (Abarenkov et al. 2024). Then, both UNITE and GenBank (v.09–2023) databases were used to determine the best match sequence (Table S1). *Suillus* genus species often exhibit diversity in their ITS regions, despite falling under the same morphological species concept, as evidenced by their fruiting body characteristics (ID: IB20050424) (Zhang et al. 2022). Consequently, in cases where different OTUs corresponded to the same match sequence, we applied annotations based on the classification of the best-matching sequence. Then, to differentiate between these OTUs, numerical labels were assigned, as exemplified by "*Suillus grevillei* 1". Assessment of host specificity for detected EcM partners of *L. decidua* (e.g., *L. porninsis*, *R. laricina*, *R. favrei*, *S. bresadolae*, *S. cavipes*, *S. grevillei*) were based on fruiting body occurrences as previously reported (Moser 1956). Then, as usually more than one OTU could be assigned to a unique MT, root tip abundances were converted to relative abundances of these OTUs based on molecular identification. For each sampling time and for each age stage within each site, proportionate abundance matrices were generated by averaging the number of root tips of each OTU across the five replicates (5 individuals for adults and young) and dividing it by the total number of root tips in each sample (600). The EcM species diversity was evaluated by richness (based on OTUs), Shannon index, and evenness α-diversity indicators. Statistical analysis of the α-indexes was performed with the nonparametric test Kruskal–Wallis when Shapiro–Wilk normality and Levene’s homogeneity of variance were not found. To analyze the relationship between observed richness and sampling time within each site and tree age, Spearman’s Rank correlation coefficient was used. To obtain estimates of actual EcM species richness, the observed species accumulation curve and jackknife second-degree estimator curve with 100 randomization with sample replacement were calculated in Estimates 9.1.0 software (Colwell and Elsensohn 2014). The variability of EcM composition among sites, tree ages, and sampling times was visualized using non-metric multidimensional scaling (NMDS). Analysis of similarity ANOSIM was used to determine if relative abundance of EcM symbionts differed between factors (sites, exposure, tree age) based on Bray–Curtis dissimilarity coefficient using vegan package (Oksanen 2011). All statistical analyses and graphs were performed in R Statistical Software (v.4.0.3; R Core Team 2021).

## Results

### EcM fungal diversity is higher in drier sites than in fresher ones

Both, adult and young European larches of all high altitudinal sites had a mycorrhization level of 100%. A total of 38 EcM morphotypes were differentiated across all four sample sites. Our molecular analysis revealed that EcM morphotyping was not suitable for distinction of taxa, as usually more than one OTU could be assigned to one EcM morphotype. Overall, we detected a total of 68 unique EcM fungal taxa after OTU clustering (Table S1). Observed species richness (based on OTUs) increased with sampling effort (sampling campaigns) in all sites and tree stages. Nevertheless, estimates of actual species richness (based on OTUs) differed from the observed species richness depending on the site, age group, and slope exposure (Fig. S1), still showing a general underestimation of species detected (Table 3).

**Table 3:** Diversity indexes for the ectomycorrhizal fungal communities associated with *L. decidua.* Richness is the total number of species (OTUs) observed within a category across different sampling times. Richness-jackknife2 is the estimated EcM species richness (OTUs) based on second order jackknife estimator with 100 randomized runs with sample replacement. Different letters indicate significant differences between factors at p < 0.05 (Kruskal–Wallis test)

Location	Tree age	Site	Richness	Richness-jackknife2	Shannon	Evenness
Schnals	Adult	North	22^a^	40	2.8^a^	0.9^a^
		South	22^a^	37	2.8^a^	0.9^a^
	Young	North	18^a^	50	2.6^a^	0.9^a^
		South	14^b^	45	2.1^b^	0.8^b^
Prettau	Adult	North	20^a^	34	2.6^a^	0.9^a^
		South	13^ab^	40	2.2^ab^	0.9^a^
	Young	North	8^b^	22	1.8^b^	0.9^a^
		South	6^b^	7	1.6^b^	0.9^a^

In general, we found higher total OTU richness in the drier and warmer Schnals compared to Prettau (χ^2^_1_ = 5.7, p = 0.0168) (Table 3). Concerning age classes, total OTU richness was higher in adult trees than in young trees, regardless of site (χ^2^_7_ = 14.7, p = 0.04). The OTU richness of adult trees differed between north- and south-exposed sites in Prettau (χ^2^_1_ = 4.1, p = 0.04), but not in Schnals (χ^2^_1_ = 0.2, p = 0.7). In the young trees, OTU richness did not differ between north- and south-exposed sites (χ^2^_1_ = 0.4, p = 0.7) regardless of location.

Over time, we found that the richness of OTUs in young trees tended to increase within the range of richness values of adult trees from the same sites, although this pattern was not significant (rho = 0.5, p = 0.7). OTU richness of young trees in the south-exposed sites remained high across sampling times.

**Fig. 2:**
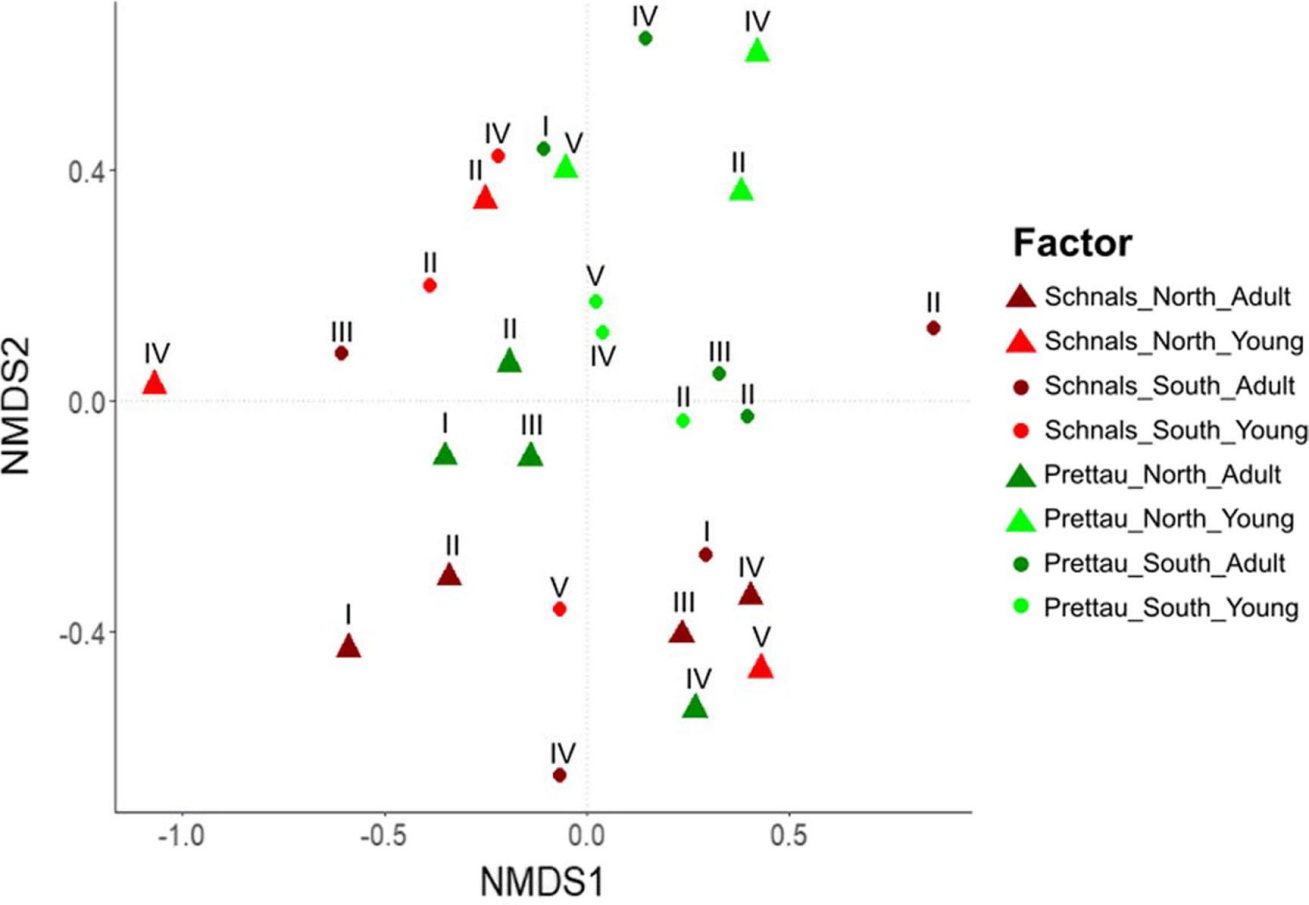
Nonmetric multidimensional scaling (NMDS) analysis based on Adonis and Bray–Curtis distance of the OTU matrix of the EcM fungal community in different *Larix* decidua sites. Stress value = 0.2389. ANOSIM each factor (region, tree age): R2 = 0.07, p = 0.006. To distinguish communities from different sampling campaigns, the sampling time is added above each sample

**Fig. 3:**
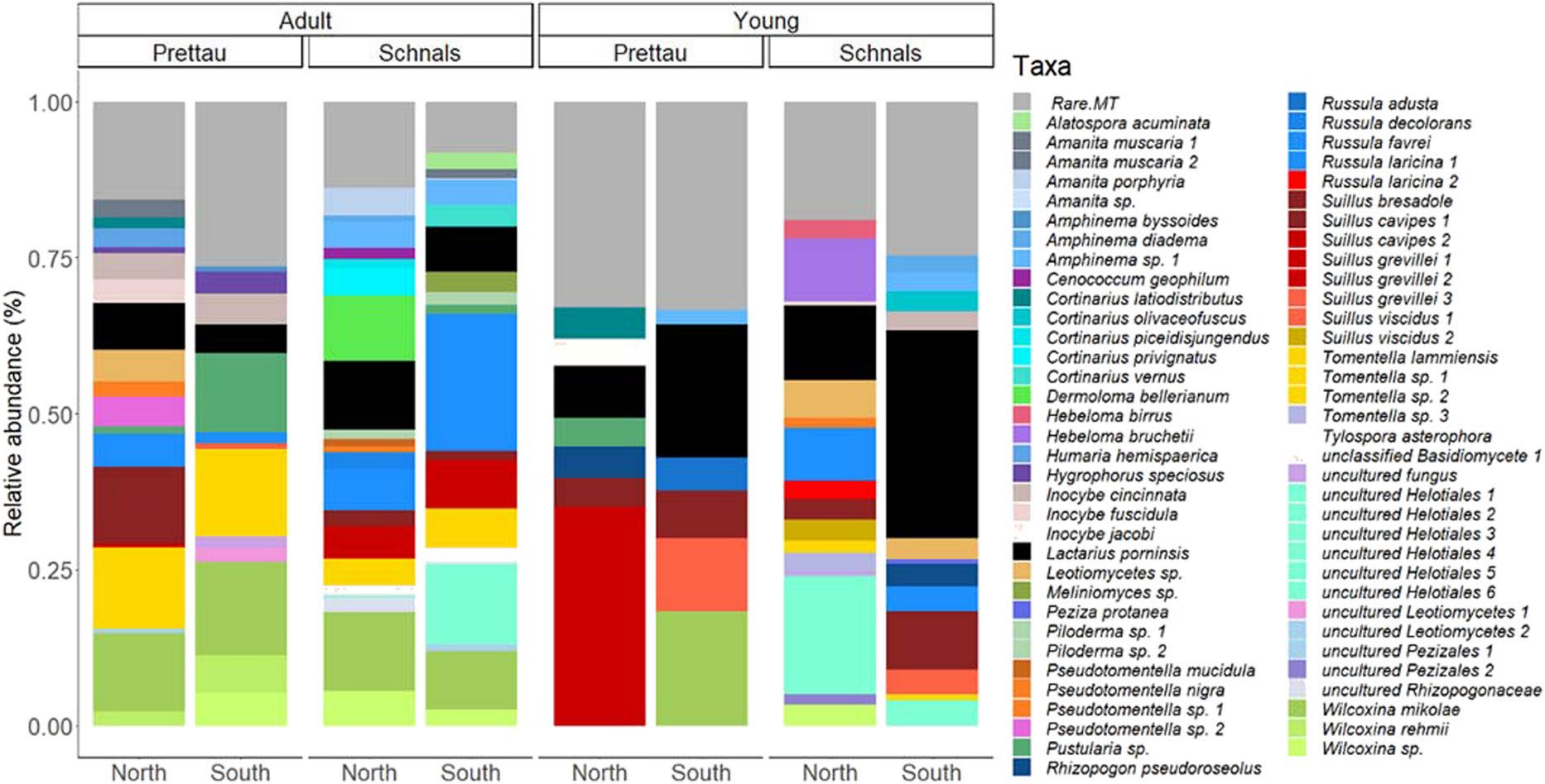
Relative abundance of EcM fungi in *Larix decidua* forests in north- or south-exposed sites across all sampling time points. The fungal composition is divided by adult and young trees. Within each tree age, the region Prettau and Schnals are further divided by their site exposure. Species belonging to the same genus are coloured in the same shades; OTUs matching to the same taxon name are coloured with the same colour. Rare MT are root tips found only once, to which no taxonomy was assigned

EcM fungal composition differed greatly among sites and between years of sampling time (Fig. 2). ANOSIM analyses based on Bray–Curtis distance matrix indicated that location (Schnals vs Prettau) and tree age (adult vs young) imposed the only effect on community structure, accounting for 14% of the variation in the EcM fungal community composition (Table S2). No other explanatory factors were significant. Nevertheless, we found that the EcM composition between the south-exposed sites of the two regions were the most similar with each other. The north-exposed site in Prettau and the south-exposed site in Schnals had the least similarities, in both tree ages. In this regard, the most evident difference in EcM composition was between young and adult trees (Fig. 3).

### Few taxa are widespread across all sites and age stages

**Fig. 4:**
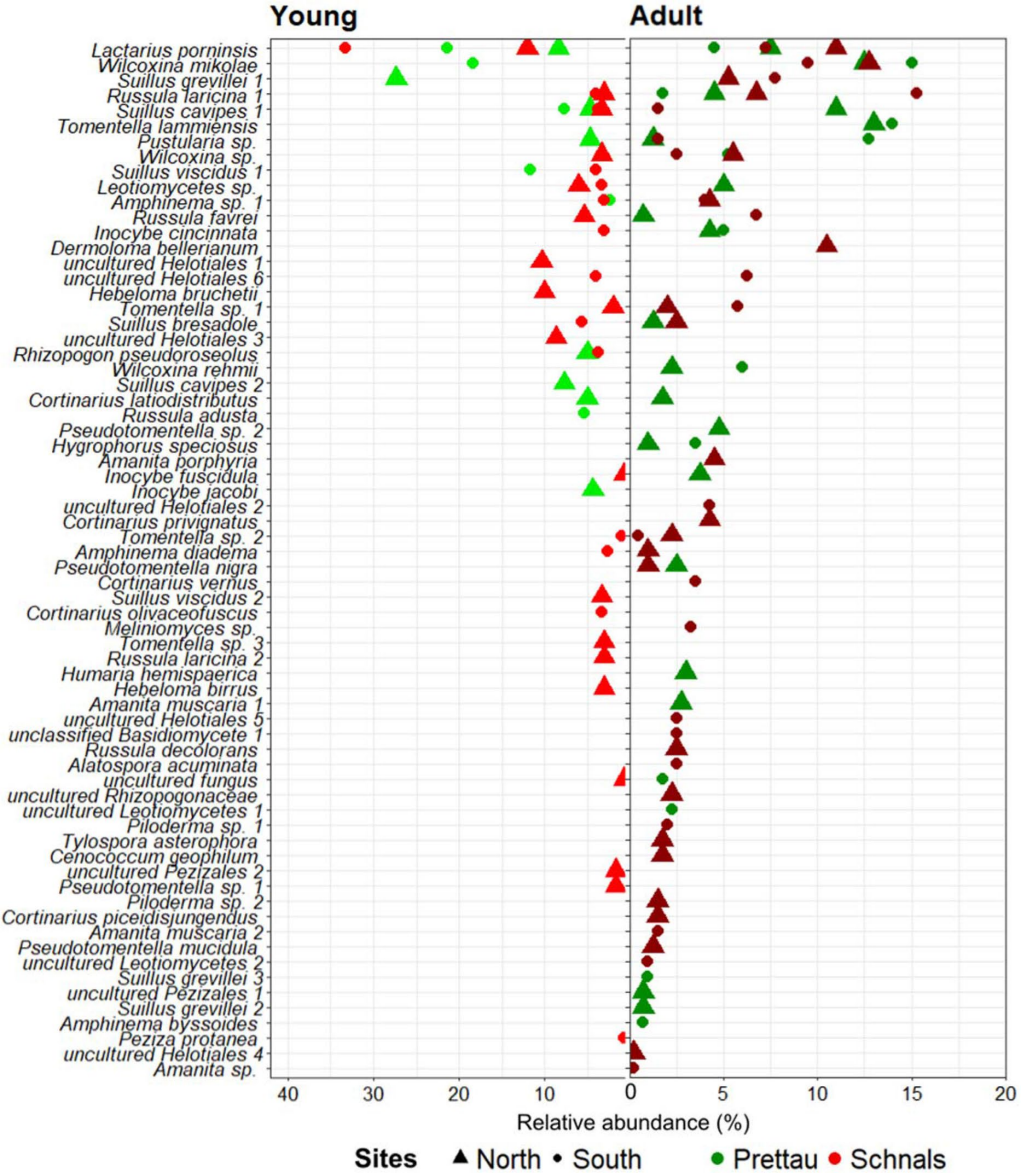
Differential representation of EcM taxa found in the roots of young and adult *L. decidua* trees. In the y-axis, EcM species are arranged based on their cumulative relative abundance across all four sites. Each species is represented by marks denoting their relative abundance (%) in the x-axis, colour-coded by location (Prettau or Schnals), and shape-coded by slope exposure (north- or south-exposed sites)

Overall, we found that Basidiomycota (70%) dominated the EcM community of both seedlings and adult *L. decidua* trees, followed by Ascomycota (30%). The EcM community belonged to a total of 17 different families, with *Suillaceae*, *Thelephoraceae*, *Russulaceae,* and *Cortinariaceae* being the most frequent. *Suillus*, *Cortinarius*, *Russula*, *Amanita*, *Pseudotomentella*/*Tomentella* were the most frequent EcM genera. We found 11 taxa as core ECM species of *L. decidua* with wide distribution: *Lactarius porninsis*, *Wilcoxina mikolae*, *Wilcoxina* sp., *Suillus cavipes* 1, *S. bresadolae*, *S. grevillei* 1, *Pustularia* sp., *Amphinema* sp. 1, *Inocybe cincinnata*, *Russula laricina* 1, and *R. favrei* (Figs. 3 and 4). These were detected as ECM in both adult and young trees, and in all regions or sites, showing a wide colonization potential. Some of these, are well known early-stage ECM fungi like *Wilcoxina mikolae*, or *L. decidua* host specialists like *L. porninsis*, *R. laricina*, *R. favrei*, *S. bresadolae*, *S. cavipes*, *S. grevillei* (Figs. 4 and S2).

Concerning age classes, we found 52 EcM taxa associated to adult trees across all sites, and 35 taxa to the young trees. Thus, species diversity of EcM fungi present on both adult and young larches was relatively high. A total of 33 taxa were unique to adult trees while 16 taxa to young trees (Fig. 4). The most abundant EcM species in adult *L. decidua* was *W. mikolae* (50%) while *L. porninsis* (75%) was the most abundant EcM partner in young trees.

When considering habitat exposure, the most frequently detected EcM partners in adult trees of the north-exposed sites in Prettau were *Tomentella lammiensis* (13%), *W. mikolae* (13%), *S. cavipes 1* (11%), and *L. pornisis* (8%). Sampling at the south-exposed site revealed *W. mikolae* (15%), *T. lammiensis* (14%), and *Pustularia* sp. (13%) to be the most abundant EcM species. In Schnals, the most frequently detected EcM taxa of adult trees of north-exposed sites were *W. mikolae* (13%), *L. pornisis* (11%), and *R. laricina* 1 (7%). In the south-exposed sites, *R. laricina 1* (15%), *W, mikolae* (10%), *S. grevillei* 1 (8%), and *L. porninsis* (7%) were the most abundant species.

In contrast to the EcM fungal community of adult trees, the most abundant mycobionts of young root trees were less diverse and dominated by few recurring taxa (Figs. 3 and 4). Naturally regenerated European larches at the north-exposed sites in Prettau were predominantly colonized by *S. grevillei* 1 (27%), *L. porninsis* (8%), and *S. cavipes* 2 (8%). In the south-exposed site, *L. porninsis* (21%), *W. mikolae* (18%), and *S. viscidus* 1 (12%) dominated on the *Larix* roots. It is worth noting that *W. mikolae* was only detected in *L. decidua* seedlings at these sites. In Schnals, *L. porninsis* (12%), uncultured Helotiales 1 and 2 (10%), and *Hebeloma bruchetii* (10%) were dominating mycorrhizal partner of the north-exposed larches. In the south-exposed sites, *L. porninsis* (33%), *S. bresadolae* (6%), and *R. laricina* 1 (4%) were the most abundant mycobionts.

Beside EcM fungi commonly found across all sites, we found many that were unique to either one region or one slope exposure. Briefly, we found 30 taxa that were uniquely found in the south-exposed sites (e.g., *R. decolorans*, *R. laricina* 2, *S. cavipes* 2, *S. viscidus* 2, *S. grevillei* 2) and 18 uniquely found in the north-exposed one (e.g., *R. adusta*, *S. grevillei 3*, *S. viscidus* 1, *Cortinarius olivaceofuscus*) (Fig. 4). In addition, 15 taxa were unique to Prettau (e.g., *Inocybe jacobi*, *S. cavipes* 2, *S. grevillei* 2, 3, *Tomentella lammiensis*, *Wilcoxina rehmii*) while 36 taxa were uniquely found in Schnals (e.g., *R. decolorans*, *R. laricina* 2, *S. viscidus* 2, *Tomentella* sp. 1, 2, 3, *Piloderma* sp.) (Fig. 4).

### The colonization by generalist EcM fungi is very dynamic

**Fig. 5:**
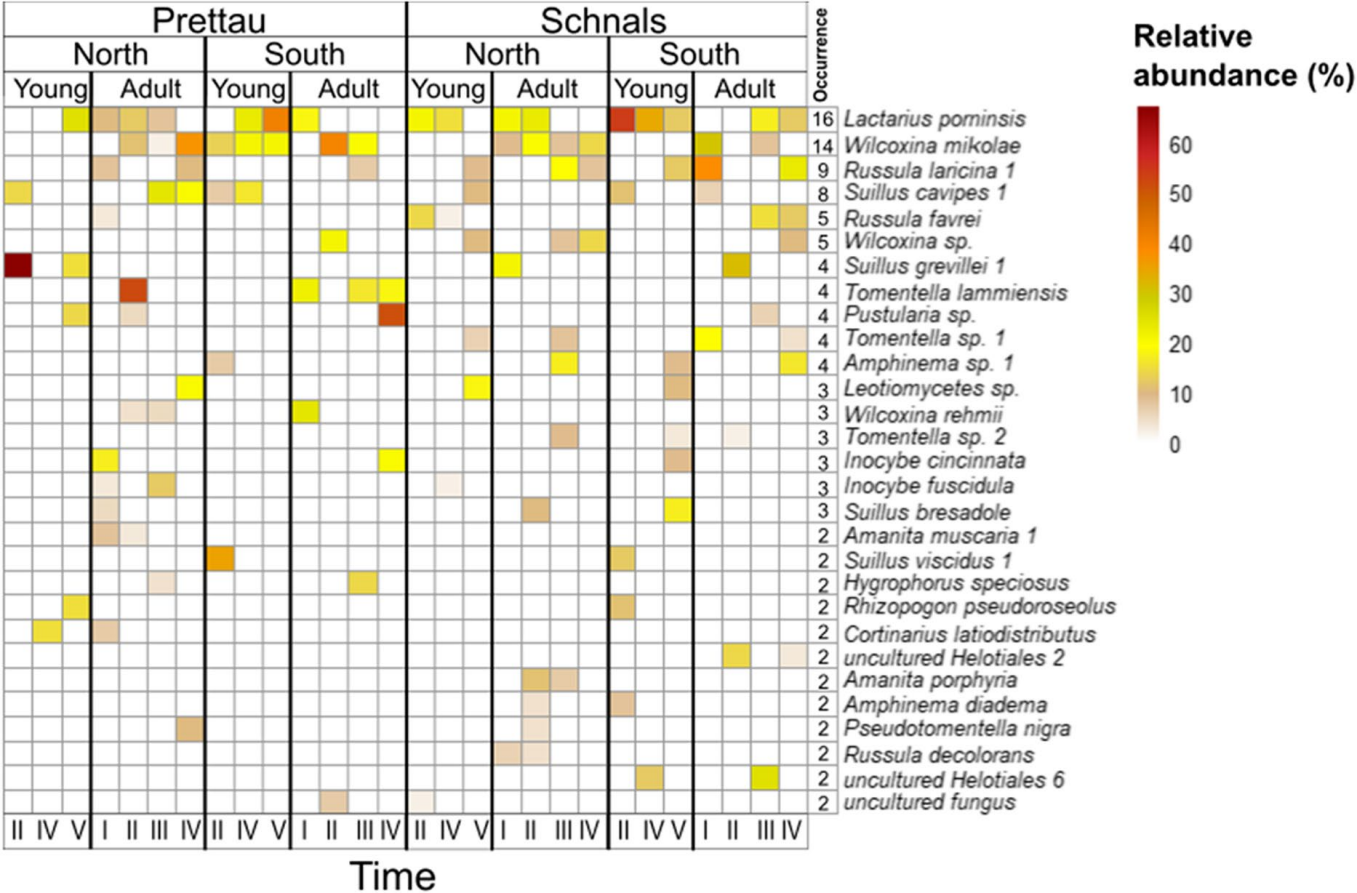
EcM fungal development across time of sampling across young and adult trees. Taxa, occurring more than once, are shown and ordered by their occurrence, with the most occurring ones, across all sites, at the top. Their relative abundance is also marked. Sampling times of young trees was always spring; sampling time of adult tree was spring (I and III) and autumn (II, IV)

The species composition of EcM fungi was remarkably homogeneous across all sample replicates within each of the four high altitudinal sites during the same sampling event, highlighting the consistency of EcM communities occurring in these specific *L. decidua* forests. However, the scenario shifted considerably between successive sampling campaigns, revealing a strong degree of temporal variability in species presence (Fig. 5). In fact, with the exception of the well-documented EcM specialists associated with *L. decidua*, many EcM fungal taxa were exclusively detected at one sampling time, showing a great dynamism of colonization and decolonization within the plant-symbiont relationship. No clear seasonal pattern could be observed in adult trees within the same year of sampling. In other words, no clear changes in EcM composition between spring and autumn could be detected. For example, *R. laricina* 1 was detected in all four sites, but at different sampling times: in the north-exposed site of Prettau at times I (spring) and IV (autumn), in the south-exposed sites at time III (spring); in Schnals, in the north-exposed site at times III and IV and in the south-exposed one at times I (spring) and IV (Fig. 5).

## Discussion

### Mycorrhizal status of natural larch stands at high altitudes

The EcM symbiosis is important for trees in natural forests at high altitudinal sites. In fact, the *L. decidua* roots investigated of both adult and young trees show a complete mycorrhization, coherent with the root colonization of naturally regenerated trees in forests and plantlets of nurseries (Leski et al. 2008; Bacher et al. 2010; Leski and Rudawska 2012). Furthermore, the species diversity of symbiotic partners of larch was relatively high (68 species) when compared to the mycobiont richness reported for other ectotrophic plants of the same altitudinal zone in the Alps with subalpine vegetation: in fact, only 20 species were reported for *Pinus cembra* forests from the same region (Mandolini et al. 2022), 28 for Swiss *Picea abies* forests (Peter et al. 2001), 39 for *Arctostaphlylos uva-ursi* (Krpata et al. 2007), and 19 for *Salix herbacea* (Mühlmann and Peintner 2008). Previous studies reported only 30 OTUs for naturally regenerated *L. decidua* in Poland (Leski and Rudawska 2012) and 23 OTUs for adult *L. kaempferi* at primary successional zones on Mount Fuji, Japan (Nara 2006). Conversely, and coherent with our findings, other *Larix* species like *L. chinensis* and *L. gmelinii* have a rich EcM fungal community of 60 and 56 OTUs, respectively (Han et al. 2017; Miyamoto et al. 2021). Both, EcM colonization rates (Nara and Hogetsu 2004) and EcM diversity (Teste et al. 2009) are higher when seedlings can develop within the common mycorrhizal network present in forest soil of mature trees. In the same way, an increased number and diversity of mycobionts present on adult trees may result from the more complex fungal community present as mycorrhizal inoculum in a mature forest. A similar mycobiont richness was detected based on fruiting body inventories in the Alpine region (Favre 1960; Horak 1963, 1985). Fruiting bodies of 50–60 potential ectomycorrhizal partners were reported in these studies, with *Suillus grevillei*, *S. viscidus* (= *S. laricinus*), *S. tridentinus,* and *Suillus (*= *Boletinus) cavipes* dominating in these fruiting body surveys. Unfortunately, there is nomenclatural confusion around *S. viscidus*: the epithets *S. laricinus*, and *S. aeruginascens* are synonyms of *S. viscidus*. *Suillus bresadolae* was first considered to be a variety of *S. viscidus*, but has now the status of a distinct species (Nguyen et al. 2016). As already reported for other studies (Kjøller 2006), fruiting body data matched only to a small extent with the actual mycorrhizal community composition detected on the roots: With the exception of *S. tridentinus*, most species detected as fruiting bodies and defined as leading EcM partner *Larix decidua* occurred as abundant mycorrhizal partners as well. However, many dominant EcM fungi do not, or only rarely, form fruiting bodies, or the fruiting bodies are inconspicuous (e.g. corticoid), hidden (on the underside of deadwood), hypogeous, or very small and thus easily overlooked (e.g., *Wilcoxina*, *Pustularia*, *Tomentella*, *Amphinema* spp.). Molecular identification of mycorrhizal partners is thus an indispensable method for obtaining a reliable picture of the actual distribution of fungi in an ecosystem. The present molecular identification of fungal symbiotic partners provided insight in the actual mycorrhizal status of European larch for the first time: *Lactarius porninsis* and *Russula laricina* 1 could be identified as ubiquitous and dominant mycorrhizal partners of larch at South Tyrol's high altitudinal sites together with *S. grevillei, S. viscidus, Pustularia* and *Wilcoxina* species. A high species richness seems to be characteristic for larches growing in such high altitudinal habitats.

Almost all dominant mycorrhizal partners of European larch growing in high altitudes are host specialists and depend on the presence of this tree species as symbiotic partner. The positive effect of mycorrhizal specialists on the establishment and development of larch is relatively well known. Particularly, larch root exudates stimulate the germination of spores from mycorrhizal partners (Ali and Jackson 1988). High abundances of mycorrhizal partners like *Suillus grevillei*, *Suillus* (=*Boletinus*) *cavipes*, and *Lactarius porninsis* result in significant increase in plant growth (Piola et al. 1995; Ohga and Wood 2000) and better nitrogen and phosphorus nutrition of mycorrhized larches compared to non-mycorrhized ones. Furthermore, *Suillus* species exhibit adaptive strategies (i.e., production of resistant sporebanks via abundant fruit bodies) ensuring the presence of EcM inoculum, even in disturbed habitats and for long time periods. Unfortunately, data about host specificities of *Pustularia* are still scarce. *Pustularia* species are part of the core larch EcM microbiome at these sites. These ascomycetes have been reported to control C transfer among different tree individuals of pines based on radioisotope labelling experiments (Cahanovitc et al. 2022). Overall, these factors are important for a successful establishment of plant hosts in environments limiting plant growth. Further studies are needed to investigate the importance of these fungi in an in-vivo EcM network.

Furthermore, the primary mycorrhizal partners of European larch generally showed no preferences for fresh or dry sites. However, there might be differently adapted populations or fungal strains: *Suillus viscidus 1* and *Suillus grevillei 3* occurred only on dry sites, and *S. cavipes 2, S. grevillei 2, S. viscidus 2, and Russula decolorans* only on fresh sites, suggesting a preference or these specific conditions. This confirms that not only *Cenococcum* (Jany et al. 2002), but also other EcM fungi have a high degree genetic diversity, enabling them to adapt to changing environmental conditions. A colonization of larch seedlings in forest nurseries with these fungi may guarantee a sustainable mycorrhization of the trees under current climate change predictions at comparable high altitudinal sites.

Previous studies have reported the importance of climate and soil characteristics on EcM fungal richness and composition at regional (Miyamoto et al. 2023) and global scales (Tedersoo et al. 2012; Bahram et al. 2021), which are even more important than geographical distance or host identity (Miyamoto et al. 2015). In our study, we observed a difference in species richness between Schnals and Prettau, with higher ECM fungal richness in warmer and drier sites compared to more humid habitats at similar altitudes (Gong et al. 2022). However, we did not find a specific EcM community composition depending either by sites or locations (i.e., climate). In other words, we did not identify a typical EcM fungal community for warmer or fresher climates at the high altitudinal sites sampled. In this regard larch clearly differs from other trees of the same area and habitat type, because *P. cembra* forests have typical EcM fungal communities (Mandolini et al. 2022). Given that larch forests are approaching their margin of distribution due to high temperatures, the increased root colonization by a more diverse set of EcM (i.e., generalists) may be seen as a chance for resilience, but it can come at the expense of specialists. This has two significant repercussions: first, generalists better adapted to warmer and drier conditions may outcompete specialists in colonizing plant host roots; Second, the incremental lack of specialists from the roots very likely reduces fungal fructification, and thus formation of soil spore banks. On the long run, this may limit the colonization success of European larches in primary succession ecosystems. The combination of these factors may facilitate the establishment of other plant communities at early stages of forest development, with potentially unknown effects on mountain forest biodiversity and ecosystems services.

### "Multi Stage" mycorrhizal partners of larches at high altitudes

Different "multi-stage" and "early-stage" mycorrhizal partners were identified regardless of location. Most specialists are “multi-stage” mycobionts. In fact, *Suillus* (= *Boletinus*) *cavipes*, *Suillus grevillei*, *S. viscidus* (= *aeruginascens*), *Lactarius porninsis,* and *Wilcoxina* spp. occurred regularly and frequently with all age classes of larches and can therefore be considered as important and typical "multi-stage" mycobionts of larches at South Tyrol's high altitudinal sites. The most frequent and important mycorrhizal partners are thus larch-specific fungi. Interestingly, *Lactarius porninsis* occurred on natural larch stands of both adult and naturally regenerated trees. Therefore, we also consider *Lactarius porninsis* as one of the most important typical "multi-stage" mycobionts of larch. The association of *L. decidua* with the larch bolete *Suillus grevillei* has long been known (Fries 1874; Melin 1922). *S. grevillei* was originally considered a mycorrhizal symbiont of older, natural stands of larch (Pachlewski 1963), but later it was also detected on the roots of larch seedlings from bare-root forest nurseries (Leski et al. 2008). We detected *S. grevillei* on naturally regenerated trees, and, altogether, it can therefore be described as a typical and important "multi-stage" mycorrhizal partner of larch. Kottke & Oberwinkler (Kottke and Oberwinkler 1988) observed that hyphal growth of *S. grevillei* is stimulated by the surface texture of the larch roots due to a specific recognition factor. Then, this species forms thick and extensive strands of hyphae (rhizomorphs) (Agerer 2001), which are used to exclusively connect plants of the same species (Kennedy et al. 2007). *Suillus viscidus* is a sister lineage of *S. bresadolae.* The latter species is adapted to high altitudes. Our study confirms that *S. bresadolae* is a distinct species, very likely with different physiological properties. *Suillus viscidus* could only be detected on the roots of adult larches in this study, but was detected in seedlings of forest nurseries in another study (Bacher et al. 2010). It can thus be considered an important “multi-stage” mycobiont of larch. Finally, *Inocybe cincinnata*, *Amphinema diadema*, *Pseudotomentella nigra*, *Russula favrei* also occurred frequently on all larch age classes, but less regularly. *Russula favrei* was first described as *Russula xerampelina* forma b from a subalpine mixed conifer forest near the Swiss National Park (Favre 1960). This form was later described as distinct species typically occurring in subalpine forest stands of *Picea abies*, *Pinus cembra*, and *L. decidua* in the alpine range (Moser 1979).

*Wilcoxina* species (e.g., *W. mikolae*) occurred on all age classes (adult, young) at the natural sites, as well as on plants from nurseries (Bacher et al. 2010). In contrast, *Wilcoxina rehmii* was detected on all larch seedlings, but not on adult larches. These results indicate that it is very important to differentiate within the genus *Wilcoxina*. Previously, it was assumed that all species of the ascomycete genus *Wilcoxina* are typical mycobiont of nursery plants (Renseigné et al. 2006), and that *Wilcoxina* does normally not occur on adult trees (Cline et al. 2005). According to our observations, the latter is true only for *W. rehmii*. The other species of the genus *Wilcoxina* are a typical and important part of the mycorrhizal community of natural sites, and are important in association with all ages of larch. Our results also contrast those of Teste et al. (Teste et al. 2009), where *W. rehmii* was detected with high abundances on adult Douglas-fir.

*Russula adusta* occurred only on adult larch plants and is typical for siliceous soils at high altitudes in association with various conifers. The larch cuttle *Hygrophorus speciosus* and *Humaria hemisphaerica* occurred only on naturally regenerated larches. *Humaria hemisphaerica* is a non-specific mycorrhizal fungus that can form mycorrhizal associations with various ectotrophic trees (e.g. *Fagus*), but also with orchids. Two other *Hygrophorus* species were reported on bare-rooted larch plants (Bacher et al. 2010), indicating that other members of this genus could also be considered as "early stage" EcM fungi.

### Factors driving temporal changes in the EcM community

This study is the first to investigate the temporal variation of EcM fungal communities in high altitudinal larch forests. The turnover of EcM partners present on the roots of a plant is subject to numerous factors, including resource availability, climatic conditions (e.g., temperature or water regimes), as well as competition among EcM for soil or plant host resources. Thus, the presence of rare species (OTUs with comparatively low relative abundance and low occurrences across time and sites) may only become evident on the host roots after environmental shifts or following perturbations, such as wildfires (Miyamoto et al. 2021). Fungal turnover rates can vary considerably. The turnover of the EcM fungal species composition on plant roots occurs within a month in oak forests (Courty et al. 2008) while EcM hyphae exhibit shorter responses, particularly following rainfalls (Allen and Kitajima 2013). We observed a nearly complete turnover of occurring rare species across sampling times. Thus, our results reveal a remarkably dynamic EcM composition. This indicates two main points: first, that there is a high mycorrhizal potential present in these habitats, and, second, that EcM fungal partner do not equally re-establish themselves on the host roots during consecutive seasons. The mycorrhizal potential of a site encompasses all mycorrhizal partners present, regardless of whether they are used for an EcM symbiosis or present as solid hyphae or resting stages (including spore banks). EcM colonization occurs from germinating fungal dispersal units (spore banks), or directly through a mycorrhizal network (Borchers and Perry 1990; Berman and Bledsoe 1998; Dickie et al. 2002). Spore banks play an extremely important role in the re-colonization of ectotrophic plants at secondary successional sites: after forest fires, for example, *Pinus muricata* seedlings were mainly colonized through spore banks of host-specific *Rhizopogon* spp. which were able to survive the fire (Baar et al. 1999; Bruns et al. 2002). It is noteworthy that pioneer plants are often associated with host-specific fungal partners with good ability to establish large spore banks (e.g., *Suilllus* spp.). Like their plant partners, these fungi follow a ruderal strategy. Furthermore, as argued for mountain beech forest (Gorfer et al. 2021), different mycorrhizal partners follow different survival and dispersal strategies: some persist in the soil and "defend" their territory for many years or decades, while others re-establish their mycelia each year (Guidot et al. 2004). Some specialists, like *Suillus grevillei*, forms extensive mycelia in the soil, which are persistently present, but highly mobile as they can "migrate" relatively quickly with their rhizomorphs (Zhou et al. 2000).

The significant number of rare species discovered in our assessment underscores the importance of temporal assessments for a thorough comprehension of EcM fungal diversity. Studies involving single sampling events only offer a snapshot in time, potentially neglecting a vast diversity of potential EcM partners and yielding inaccurate conclusion about the structural and functional potential of an ecosystem. Unfortunately, the driving forces behind root colonization by different EcM fungi, causing community-level turnover, remain largely unknown.

## Conclusions and outlook

This is the first study addressing the mycorrhizal status of natural *Larix decidua* forests at high altitudes, revealing the significance of the EcM symbiosis for both adult and young larch trees, as the roots were always completely mycorrhized. The symbiotic partners of larch exhibited a remarkable diversity, with 68 identified species, surpassing the mycobiont richness reported for other ectotrophic plants in similar habitats of the Alps. Our molecular identification of fungal symbiotic partners, a first for European larch in natural habitats, highlighted the importance and ubiquitous mycorrhizal partners such as *Lactarius porninsis*, *Russula laricina*, *Suillus* (= *Boletinus*) *cavipes, S. grevillei*, *S. viscidus, Pustularia* and *Wilcoxina spp.*. These dominant mycorrhizal partners play crucial roles in enhancing plant growth and nutrition. Furthermore, the study identified different mycorrhizal partners that varied across age classes, emphasizing the importance of considering the multi-stage mycorrhizal associations for mycorrhizal inoculations. Our findings also contribute valuable insights into factors influencing temporal changes in EcM communities. The dynamic nature of EcM composition, including the existence of rare species, underscores the importance of temporal assessments for a comprehensive understanding of EcM fungal diversity.

Future research could focus into understanding the ecological and environmental factors influencing the temporal dynamics of EcM communities. Furthermore, investigating the specific physiological properties of distinct EcM species, particularly those adapted to high altitudes, could enhance our understanding of their roles and interactions within the symbiotic relationships. A better understanding of the physiological and functional properties of ectomycorrhizal partners would form a solid base for a future modelling of the potential impacts of climate change on EcM communities and thus also on host plant performance. In vivo environmental variable manipulation experiments (temperature, CO_2_, drought) or in vitro studies assessing the physiological range and effect of selected EcM partner on the host plant performance could help to provide such information. Long-term monitoring studies could also provide valuable insights into how EcM communities respond to climate variability and change over extended periods. The observed differences in EcM fungal richness between warmer and drier sites compared to more humid habitats at similar altitudes emphasize the need for further exploration in this direction.

## Supplementary Information

Below is the link to the electronic supplementary material.

Supplementary file1 (PDF 489 KB)

## Data Availability

The raw sequencing data were deposited in GenBank SRA database (https://www.ncbi.nlm.nih.gov/genbank/) under the accession numbers referenced in the Supplementary Material Table S1.
