# Supplementary material for: Ectomycorrhizal communities of adult and young European larch are diverse and dynamics at high altitudinal sites
Source: Plant Soil. 2024 May 21;506(1-2):691–707. doi: 10.1007/s11104-024-06721-8 (PMC11839878; doi:10.1007/s11104-024-06721-8)
Supplement: Supplementary file 1 — Supplementary file1 (PDF 489 KB) [file 11104_2024_6721_MOESM1_ESM.pdf]

Ectomycorrhizal communities of adult and young European larch are diverse and dynamics at high altitudinal sites

SUPPLEMENTARY MATERIAL

Table S1 Fungal ITS sequence identification and their accession numbers (GenBank SRA database). The taxonomic affiliation and bootstrap values of each unique sequence retrieved by UNITE database (using kmers analysis with 1000 interactions) are reported. Best match sequence based on GenBank database with the relative identity percentage are also reported.

**Taxon**	**Accession No.**	**Taxonomic affiliation (bootstrap %)***	**Best match species****	**Best match** accession No.**	**Identity (%)**
Alatospora acuminata	HM044524	Alatospora unclassified (83)	Alatospora acuminata	MK353088	97%
Amanita muscaria 1	HM044482	Amanita muscaria (99)	Amanita muscaria	MT351050	98%
Amanita muscaria 2	HM044487	Amanita muscaria (96)	Amanita muscaria	MT351050	99.84
Amanita porphyria	HM044539	Amanita porphyria (100)	Amanita porphyria	JF899548	99%
Amanita sp.	HM044522	Unclassified Amanita sp. (100)	Amanita sp.	MN336274	95%
Amphinema byssoides	HM044498	Amphinema byssoides (97)	Amphinema byssoides	LR694190	97%
Amphinema diadema	HM044547	Unclassified Amphinema sp. (99)	Amphinema diadema	GQ162811	96%
Amphinema sp. 1	HM044546	Unclassified Amphinema sp. (99)	Amphinema sp. 1		Locked
Cenococcum geophilum	HM044569	Cenococcum unclassified (99)	Cenococcum geophilum	OQ418519	99%
Cortinarius latiodistributus	HM044511	Cortinarius anomalus (95)	Cortinarius latiodistributus	MZ663793	99%
Cortinarius olivaceofuscus	HM044544	Cortinarius cinnamomeus (100)	Cortinarius olivaceofuscus	AY669585	99.75
Cortinarius piceidisjungendus	HM044551	Cortinarius orasericeus (100)	Cortinarius piceidisjungendus	FJ717584	98%
Cortinarius privignatus	HM044561	Cortinarius cf. privignatus (100)	Cortinarius privignatus	KP165569	99%
Cortinarius vernus	HM044540	Cortinarius decipiens (99)	Cortinarius vernus	KY964770	98%
Dermoloma bellerianum	HM044480	Unclassified Dermoloma sp. (84)	Dermoloma bellerianum	MW193805	98.92
Hebeloma birrus	HM044476	Hebeloma theobrominum (100)	Hebeloma birrus	KJ146710	96%
Hebeloma bruchetii	GU181869	Hebeloma psammophilum (98)	Hebeloma bruchetii	AY948195	99%
Humaria hemispaerica	HM044499	Humaria hemisphaerica (100)	Humaria hemispaerica	DQ200832	99%
Hygrophorus speciosus	HM044495	Hygrophorus speciosus (100)	Hygrophorus speciosus	DQ097884	99%
Inocybe cincinnata	HM044545	Inocybe cincinnata (100)	Inocybe cincinnata 1	MW845946	99%
Inocybe fuscidula	HM044564	Inocybe fuscidula (100)	Inocybe fuscidula	MH930194	99%
Inocybe jacobi	HM044531	Unclassified Inocybe sp. (91)	Inocybe jacobi	HQ604811	89%
Lactarius porninsis	HM044509	Lactarius deliciosus_var_olivaceosordidus (95)	Lactarius porninsis	MT302585	99%
Leotiomycetes sp.	HM044486	Unclassified Helotiales (90)	Leotiomycetes sp.	KX909226	93%
Meliniomyces sp.	HM044523	Unclassified Meliniomyces sp. (99)	Meliniomyces sp. 1	KC007335	98%
Peziza protanea	HM044521	Peziza sp. (95)	Peziza protanea	KC243960	96%

Piloderma sp. 1	HM044543	Unclassified Piloderma sp. (85)	Piloderma sp. 2	FJ236851	85%
Piloderma sp. 2	HM044557	Piloderma sphaerospermum (100)	unclassified Basidiomycete 2		Locked
Pseudotomentella mucidulus	HM044485	Pseudotomentella mucidulus (100)	Pseudotomentella mucidulus	MK290725	99%
Pseudotomentella nigra	HM044549	Unclassified Pseudotomentella sp. (90)	Pseudotomentella nigra	MK290719	99%
Pseudotomentella sp. 1	HM044464	Polyozellus medius (86)	Pseudotomentella sp. 1	MK290713	94%
Pseudotomentella sp. 2	HM044465	Polyozellus medius (100)	Pseudotomentella sp. 2	MK290713	94%
Pustularia sp.	HM044534	Sepultariella semi-immersa (98)	Pyronemataceae 1	EU726302	98%
Rhizopogon pseudoroseolus	HM044502	unclassified (100)	Rhizopogon pseudoroseolus	MN737836	84%
Russula adusta	HM044555	Russula adusta (100)	Russula adusta	KX267635	100%
Russula decolorans	HM044550	Russula decolorans (100)	Russula decolorans	MN992510	100%
Russula favrei	HM044477	Russula favrei (99)	Russula favrei	KU205324	99%
Russula laricina 1	HM044475	Russula cessans (99)	Russula laricina 1	JN944008	99%
Russula laricina 2	HM044548	Russula cessans (98)	Russula laricina 2	JN944008	99%
Suillus bresadolae	HM044567	Suillus bresadolae (98)	Suillus viscidus 2	KU721449	99.6
Suillus cavipes 1	HM044566	Suillus cavipes (70)	Suillus cavipes 1	MT302581	70%
Suillus cavipes 2	HM044532	Suillus cavipes (78)	Suillus cavipes 2	MT302581	80%
Suillus grevillei 1	HM044473	Suillus gravillei (100)	Suillus grevillei 1	HM347659	100%
Suillus grevillei 2	HM044478	Suillus grevillei (94)	Suillus grevillei 2	KU721469	82%
Suillus grevillei 3	HM044494	Suillus grevillei (94)	Suillus grevillei 3	OR242706	94%
Suillus viscidus 1	HM044484	Suillus viscidus (92)	Suillus viscidus 1	OR242707	95%
Suillus viscidus 2	HM044467	Suillus viscidus (97)	Suillus viscidus 3	OR242702	97%
Tomentella lammiensis	HM044490	Unclassified Tomentella sp. (100)	Tomentella lammiensis	MG136841	99%
Tomentella sp. 1	HM044535	Tomentella spinosispora (99)	Tomentella sp. 1	MN047052	99%
Tomentella sp. 2	HM044529	Unclassified Tomentella sp. (97)	Tomentella sp. 2	LC547726	98%
Tomentella sp. 3	HM044553	Unclassified Tomentella sp. (100)	Tomentella sp. 3	AJ534913	99%
Tylospora asterophora	HM044466	Tylospora asterophora (89)	Tylospora asterophora	JQ711977	89%
unclassified Basidiomycete 1	HM044508	Unclassified Basidiomycete (100)	unclassified Basidiomycete 1		92%
uncultured *fungal* sp.	HM044556	unclassified (100)	uncultured fungal sp.	EF434097	83%
uncultured Helotiales 1	HM044497	Unclassified Meliniomyces (95)	uncultured Helotiales 1		96%
uncultured Helotiales 2	HM044528	Ascomycota unclassified (83)	uncultured Helotiales 1		Locked
uncultured Helotiales 3	HM044526	Unclassified Meliniomyces (98)	uncultured Helotiales 2		92%
uncultured Helotiales 4	HM044588	Uncultured Helotiales sp. (97)	uncultured Helotiales 3		98%

uncultured Helotiales 5	HM044483	Thelephoraceae unclassified (100)	uncultured Helotiales 4		96%
uncultured Helotiales 6	HM044552	Unclassified Helotiales (98)	Vestigium trifidum	KC407777	97%
uncultured Leotiomycetes 1	HM044506	Cadophora luteo-olivacea (91)	uncultured Leotiomycetes 1		92%
uncultured Leotiomycetes 2	HM044542	Ascomycota unclassified (91)	uncultured Leotiomycetes 2		83%
uncultured Pezizales 1	HM044525	Ascomycota unclassified (99)	uncultured Pezizales 1		92%
uncultured Pezizales 2	HM044527	Ascomycota unclassified (100)	uncultured Pezizales 1		99%
uncultured Rhizopogonaceae	HM044496	Ascomycota unclassified (93)	uncultured Rhizopogonaceae		78%
Wilcoxina mikolae	GU181847	Wilcoxina mikolae (100)	Wilcoxina mikolae	KU061020	99%
Wilcoxina rehmii	HM044510	Wilcoxina rehmii (100)	Pyronemataceae 2	EF611145	91%
Wilcoxina sp.	HM044479	Ascomycota unclassified (100)	Wilcoxina 2	AJ893249	98%

*Best sequence match obtained from the UNITE+INSD database (v.18-07-2023).
**Best sequence match obtained from the GenBank database (09-2023).

Table. S2 Statistical differences (ANOSIM) in variance between EcM community compositions between regions based on Bray-Curtis distance matrix.

	Df	SumOfSqs	R2	F	Pr(>F)
Region	1	0.5659	0.07006	1.9588	0.006
Tree.age	1	0.5659	0.07002	2.0367	0.016
Residual	25	6.9456	0.85994		
Total	27	8.0765	1.0000		

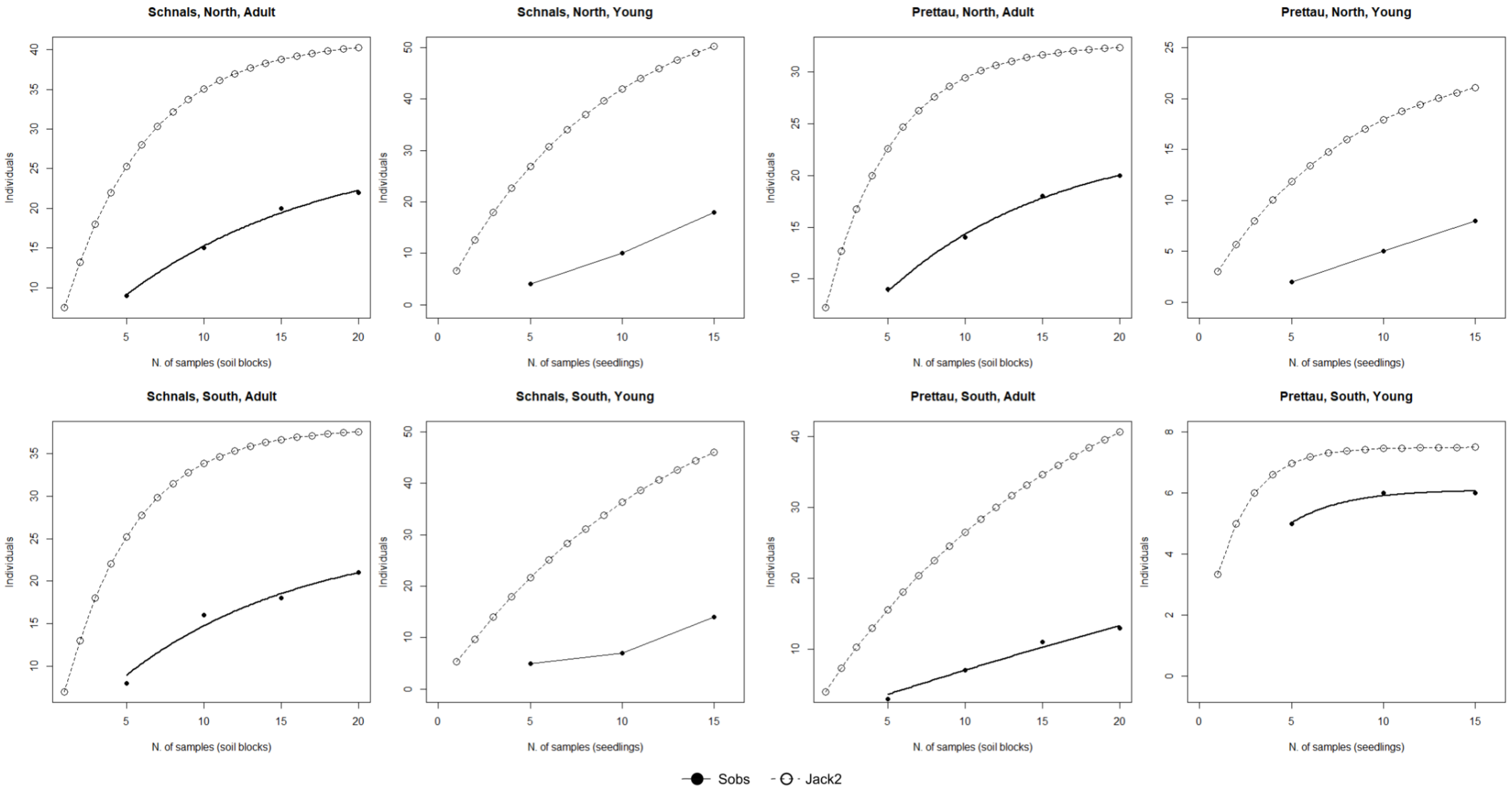

Figure S1 Ectomycorrhizal species richness estimation curves for seedlings (young) and adult *Larix decidua* trees. Curves are reported for each location (Schnals and Prettau), each slope-exposure (north- and south-exposed), and each tree age (young and adult). Within each sampling campaign (3, young; 4 adults), 5 seedlings (tot=15) or 5 soil blocks (tot=20) were sampled for young and adult trees, respectively. Sobs, species observed (OTUs); Jack2, second order jackknife estimator (100 randomized runs with sample replacement were used).

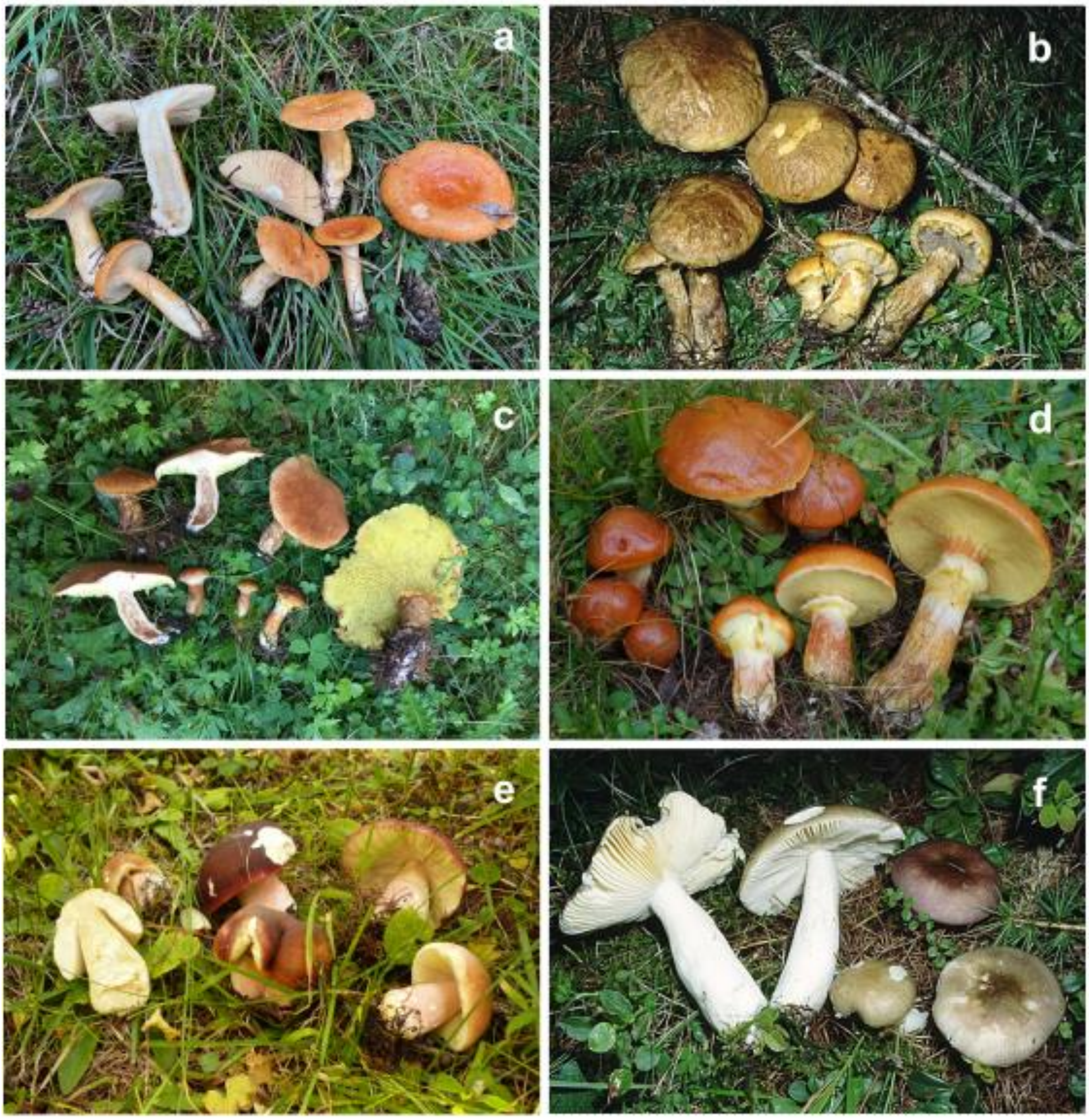

Figure S2 Fruiting body of the EcM fungal specialist of *Larix decidua*. (a) *Lactarius porninsis*; (b) *Suillus bresadolae*; (c) *Suillus cavipes*; (d) *Suillus grevillei*; (e) *Russula favrei*; (f) *Russula laricina*.
